# Insufficient evidence for non-neutrality of synonymous mutations

**DOI:** 10.1038/s41586-023-05865-4

**Published:** 2023-04-19

**Authors:** Leonid Kruglyak, Andreas Beyer, Joshua S. Bloom, Jan Grossbach, Tami D. Lieberman, Christopher P. Mancuso, Matthew S. Rich, Gavin Sherlock, Craig D. Kaplan

**Affiliations:** 1 Department of Human Genetics, University of California, Los Angeles, Los Angeles, CA, USA.; 2 Department of Biological Chemistry, University of California, Los Angeles, Los Angeles, CA, USA.; 3 Howard Hughes Medical Institute, Chevy Chase, MD, USA.; 4 Cluster of Excellence on Cellular Stress Responses in Age-associated Diseases (CECAD), University of Cologne, Cologne, Germany.; 5 Institute for Genetics, Faculty of Mathematics and Natural Sciences, University of Cologne, Cologne, Germany.; 6 Institute for Medical Engineering and Sciences, Massachusetts Institute of Technology, Cambridge, MA, USA.; 7 Department of Civil and Environmental Engineering, Massachusetts Institute of Technology, Cambridge, MA, USA.; 8 Department of Biology, University of Utah, Salt Lake City, UT, USA.; 9 Department of Genetics, Stanford University, Stanford, CA, USA.; 10 Department of Biological Sciences, University of Pittsburgh, Pittsburgh, PA, USA.

Many lines of evidence accumulated over decades of research have shown that, because they do not lead to sequence changes in proteins, synonymous mutations are much less likely than nonsynonymous ones to have biological effects^1^, although exceptions exist^2–4^. In a recent paper^5^, Shen et al. claimed that most synonymous mutations in the coding regions of 21 yeast genes were deleterious. We argue that, owing to technical issues with the experimental design and replication, this claim is not supported by the data reported by Shen et al.^5^.

Three critical issues preclude drawing any conclusions about the typical effects of synonymous variants on fitness on the basis of the data reported by Shen et al.^5^. First, there is an absence of appropriate matched wild-type (WT) controls for strain background with which to compare variant effects. Second, technical replicates are treated as biological replicates in competition-based fitness assays. As a consequence, potential biological variation in fitness is not captured, rendering statistical analysis moot. Third, the lack of biological replicates in independent growth assays intended to validate competition-based fitness measurements precludes the interpretation that most synonymous variants are deleterious in yeast.

Shen et al.^5^ set out to examine fitness effects of synonymous and nonsynonymous mutations in a curated set of 21 genes in yeast^5^. They first used CRISPR–Cas9 to create 21 strains, each carrying a deletion of 150 nucleotides and a common sgRNA target in one of the genes. They then used CRISPR–Cas9 to generate double-stranded breaks at the deletion sites, which were repaired with pools of synthesized templates homologous to sequences flanking each deletion and carrying each of the 450 possible single-nucleotide variants in the chosen 150-nucleotide region of each gene. Shen et al.^5^ then subjected the resulting strain libraries to competitive growth, with a single WT strain, derived from the repair of a strain carrying a deletion in one of the genes (*ASC1*), mixed into the libraries as a control.

Crucially, the repair template pools used by Shen et al.^5^ did not contain any WT sequences that would enable WT versions of each gene to be created in a manner identical to, and in parallel with, the creation of genome-edited strains. Inclusion of such WT strains in the libraries would have controlled for background effects on fitness specific to each edited strain. Such WT controls would be derived at the same time, and from the same colony or colonies, as the mutant pools. While we detail several specific possibilities that underscore why controlling for strain background is essential, there may also be uncontrolled strain effects beyond those that we consider below.

First, transformation of yeast itself can be mutagenic^6,7^, and two rounds of CRISPR–Cas9 editing could generate off-target effects, with different guide RNAs causing different types of off-target effects and with different frequencies. These experimental manipulations mean that each genetically engineered deletion strain represents a unique genetic background potentially carrying unascertained mutations that may affect strain fitness (Fig. 1). Second, the effects of deleting particular genes may have promoted the occurrence of additional mutations or may have precipitated the selection of growth-defect-suppressing mutations before rescue with the variant library. For example, deletion of *RAD6* is expected to impair DNA repair and may lead to chromosomal rearrangements^8^, deletion of *EST1* is expected to alter telomere length and function^9,10^ and deletion of *PAF1* is expected to cause defects in multiple histone modifications^11,12^. Thus, it cannot be assumed that such genetic backgrounds would automatically be complemented to WT fitness after reintroduction of a WT or neutral variant. These effects, as well as additional uncontrolled effects, could make neutral variants appear deleterious. Differences between the 21 genetic backgrounds could have been examined with whole-genome sequencing of edited strains, but such sequencing was not performed.

Second, further unascertained fitness-altering mutations may arise during CRISPR–Cas9-mediated insertion of variant alleles, contributing to the observed fitness differences among different edits of each gene. Critically, in pooled library construction, each individual variant derives from many colonies, and these colonies sample a distribution of fitness effects arising from any variant-independent mechanisms; the reported fitness for each variant would reflect the average of this distribution (Fig. 1 (bottom)). By contrast, the fitness of an individual WT colony selected as the control would not represent this average. Instead, selection of a single WT strain is especially sensitive to potential ascertainment bias, as any obviously unhealthy strain would not be selected but, within the variant pools and among pools of the same variant, there is no such filter. This ascertainment bias could have been avoided if matched WT strains for each gene were generated for each mutant pool. Furthermore, biological replicates with independent derivation of edited pools, along with technical replicates of competition for each biological replicate, would enable true measures of variant fitness and of strain handling or generation effects.

Third, we argue that the monoculture growth assay as carried out by Shen et al.^5^ is insufficient to validate the observed fitness defects. Shen et al.^5^ reconstructed a set of mutants and measured their growth rates in a non-pooled approach (figure 1d of ref. 5). We note that the selection of mutants was biased towards those with larger fitness effects in the pooled assays (median fitness of all generated variants = 0.988; median fitness of those selected for reconstruction = 0.951). Whereas the results in figure 1d of Shen et al.^5^ recapitulate those of the competition-based assay for variants with large effects, the assay was not constructed in a way to recapitulate small effects. When only variants with a sequencing-based fitness of ≥0.981 are considered (75% of all generated missense and synonymous variants have a measured fitness effect smaller than this), the average growth-based fitness of the reconstructed strains clusters around 1 (median = 0.997). Although these results do not disprove the reported distribution of fitness effects, they provide insufficient validation for small effects. The authors did not measure potential biological variation among independently isolated strain replicates and did not include matched WT controls. Direct demonstration that the monoculture growth assay can distinguish fitness defects on the order of 1–3%, and that independently generated strains that are supposed to be identical do not differ from each other in fitness by a similar amount, is essential for validity of any conclusions based on this assay.

In summary, the following key considerations apply to any experiment comparing the effects of synonymous and nonsynonymous variants on yeast fitness. Growth measurements for multiple independent isolates of each deletion are needed to enable the determination of potential off-target effects or deletion-specific fitness effects. Whole-genome sequencing of each isolated deletion strain should be performed. Biological replicates should be employed during the creation of variant libraries by genome editing to reliably measure the fitness effect, if any, for each variant; otherwise, these cannot be distinguished from off-target effects during library construction. As a confirmatory step, direct fitness measurements of multiple independently created individual variants of a gene, in parallel with WT alleles created on the identical genetic background, should be performed. More generally, we note that careful consideration of potential sources of experimental variability should be coupled, whenever possible, with direct experimental testing of assumptions that might otherwise rely solely on theoretical arguments. Such practices could allow for greater confidence in ruling out alternative explanations.

## Figures and Tables

**Fig. 1 |:**
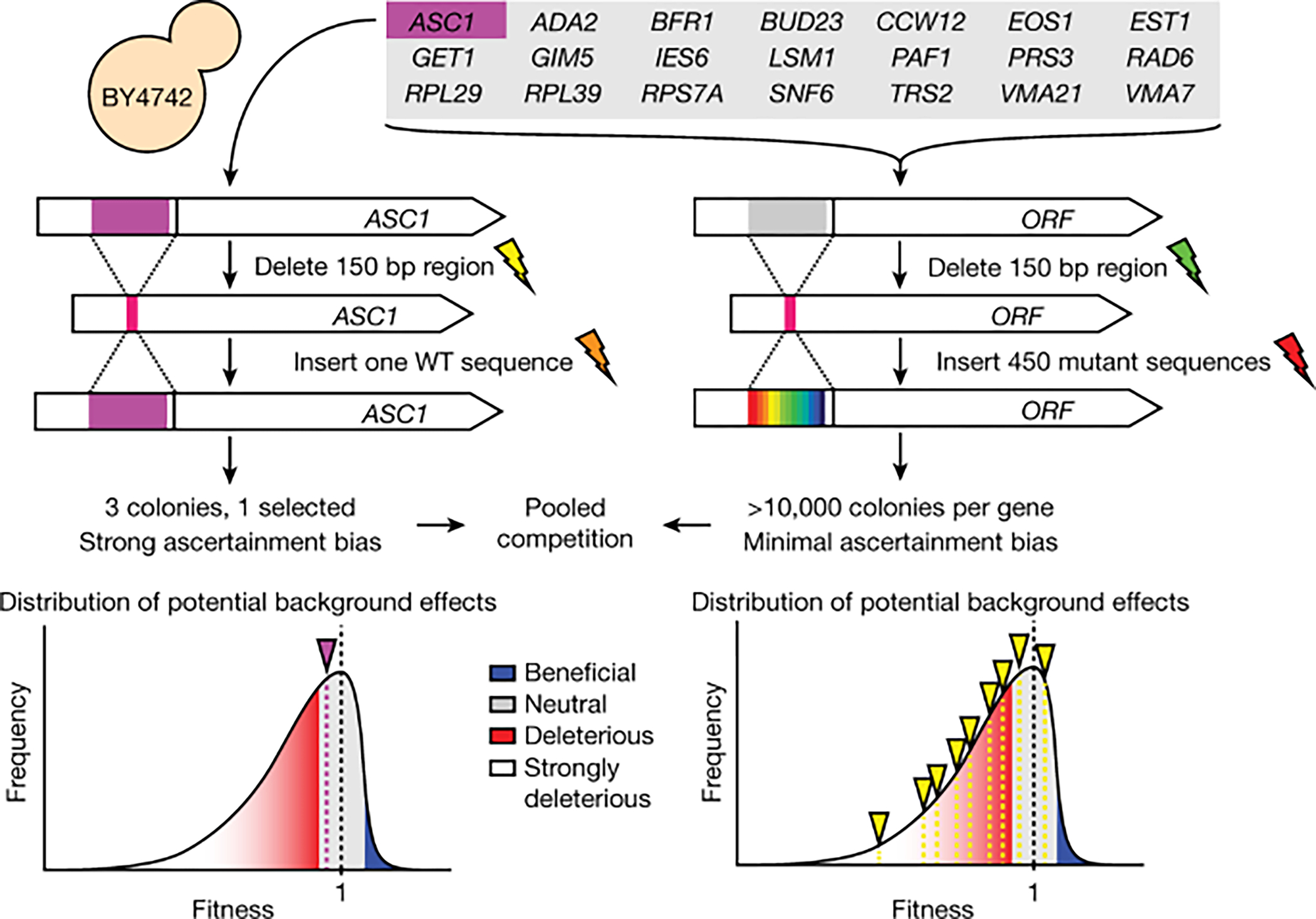
Schematic of the approach taken by Shen et al. for strain creation. Parallel strain construction can result in background effects on fitness (indicated by lightning bolts) that may be different between strains (yellow bolt versus green bolt for the first round of editing; orange versus red for the second round of editing). Differences in background effects for individual clones may be reflected by a fitness distribution (bottom). Selection of a single WT strain (bottom left) will probably come from the healthy part of the distribution, whereas mutant variants will represent the average of background effects across the distribution (bottom right) reflecting possible ascertainment bias of unmatched WT strain construction.

## Data Availability

Data discussed are from Shen et al.^5^.
